# Color Biomimetics in Textile Design: Reproduction of Natural Plant Colors through Instrumental Colorant Formulation

**DOI:** 10.3390/jimaging10070150

**Published:** 2024-06-21

**Authors:** Isabel Cabral, Amanda Schuch, Fernanda Steffens

**Affiliations:** 1 Centre for Textile Science and Technology (2C2T), University of Minho, 4800-058 Guimarães, Portugal; 2 Department of Textile Engineering, Textile Engineering Post Graduate Program (PGETEX), Federal University of Santa Catarina, Blumenau 89036-256, Brazil; amanda.schuch@posgrad.ufsc.br (A.S.); fernanda.steffens@ufsc.br (F.S.)

**Keywords:** biomimicry, colorimetry, design, textile, dyeing, spectral reflectance

## Abstract

This paper explores the intersection of colorimetry and biomimetics in textile design, focusing on mimicking natural plant colors in dyed textiles via instrumental colorant formulation. The experimental work was conducted with two polyester substrates dyed with disperse dyes using the exhaustion process. Textiles dyed with different dye colors and concentrations were measured in a spectrophotometer and a database was created in Datacolor Match Textile software version 2.4.1 (0) with the samples’ colorimetric properties. Colorant recipe formulation encompassed the definition and measurement of the pattern colors (along four defined natural plants), the selection of the colorants, and the software calculation of the recipes. After textile dyeing with the lowest expected CIELAB color difference (ΔE*) value recipe for each pattern color, a comparative analysis was conducted by spectral reflectance and visual assessment. Scanning electron microscopy and white light interferometry were also used to characterize the surface of the natural elements. Samples dyed with the formulated recipe attained good chromatic similarity with the respective natural plants’ colors, and the majority of the samples presented ΔE* between 1.5 and 4.0. Additionally, recipe optimization can also be conducted based on the colorimetric evaluation. This research contributes a design framework for biomimicking colors in textile design, establishing a systematic method based on colorimetry and color theory that enables the reproduction of nature’s color palette through the effective use of colorants.

## 1. Introduction

Nature has long been a source of inspiration for textile and fashion designers, particularly through vibrant hues, color harmonies, and intricate patterns. The definition of a color palette is a pivotal step in the design process, and the approach toward reproducing the chromatics of nature can encompass different methods. This research focuses on the biomimicry of pigmentary colors in dyed textiles based on colorimetry principles, considering color measurement and instrumental colorant formulation.

Biomimicry and biomimetics are interdisciplinary fields that utilize nature as a model and mimic or take inspiration from it to address contemporary challenges [1,2]. This growing field of research has been boosted by advances in biology and engineering, and its potential to promote innovation and sustainability has been highlighted [3,4]. Biomimetics has become increasingly relevant to textile design and engineering owing to the added value of textiles reproducing nature aesthetics and/or functional properties, such as mimicking the ability of lotus leaves to repel water in self-cleaning superhydrophobic textiles, the shark skin effect in swimsuits to reduce frictional resistance and enhance hydrodynamics, as well as diverse chromatic effects [5,6,7,8].

Color biomimetics in textiles encompasses diverse approaches, spanning from pigmentary to structural coloration, dynamic camouflage, and bioluminescence [9,10,11]. Pigmentary coloration refers to a photochemical process involving selective absorption of incident light, whereas in structural coloration, the process is photophysical, and color depends on how incident light interacts with the physical structure [12,13,14]. The mechanisms of structural colors found in nature, such as beetles and opals, have been mimicked in textiles, namely through the synthesis and deposition methods of photonic crystals in substrates [15,16]. Additionally, structural colors can exhibit an iridescent effect, meaning that colors depend on the angle of vision or illumination. Recent research has studied how structural colors and respective iridescence are affected by textile substrate morphology and fiber type, highlighting their potential for sustainable design [17].

Natural camouflage mechanisms, such as those observed in chameleons and cephalopods, have also been inspiring textile applications with dynamic textile coloration [9]. Researchers have explored the use of color change materials that reversibly react to different stimuli, such as thermochromic, photochromic, and hydrochromic colorants [18,19,20], a combination of photochromic pigments and structural colors [21], camouflage patterns with thermochromic leuco dyes [22], and in combination with liquid crystals [23] and camouflage patterns in thermal vision [24].

Furthermore, the phenomenon of bioluminescence, observed in certain organisms that emit light as a result of chemical reactions, has inspired innovative approaches in interactive textiles [25]. Light-emitting materials such as phosphorescent or fluorescent pigments, luminescent yarns or threads, and light-emitting diodes (LEDs) have been integrated into textiles through different processes [26], allowing the creation of creative expressions and visual impact.

Defining an effective color palette encompasses the creation of a set of coordinating colors selected to work together, which is a crucial step in fashion and textile design, involving a creative and systematic approach through inspiration, selection, specification, and evaluation phases [27,28]. Color inspiration can arise from various sources that can be directly linked to colors designers have collected or observed in their daily lives, such as in nature, artifacts, and digital media. Alternatively, it can be guided by conceptualization, focusing on colors that evoke a certain mood or theme [29,30].

Based on their inspiration, designers must carefully explore and select a cohesive set of colors that will compose the color palette for a particular project or collection. This process involves researching color steered by principles of color theory, trends, and practical experimentation, namely using colored materials such as inks and pencils, swatches of colored paper or fabrics, software programs that allow picking colors with an eyedropper tool from a selected digital image, established visual color systems such as Pantone, Munsell Color System, or Natural Color System, online applications, and/or color forecasting through websites or reference magazines that present information on color trends [28,31,32,33]. The wide range of options depends on the designer’s preferences, accessibility, and specific project requirements.

Color boards or swatch books are often created to document color selection, whereas reliable strategies should be employed for effective color communication with manufacturing supply chains and clients [27]. For this purpose, visual and numerical color specification systems are used, such as Pantone, Munsell Color System, CMYK (Cyan, Magenta, Yellow, and Black system), and other systems that allow associating a numerical code to a selected color or color swatch [34,35].

Colorimetry, as a discipline focused on quantifying and physically describing color, assumes an important role in this process, enabling color specification, color difference evaluation, and the prediction of color appearance [36]. By using instrumental methods and standardized color space systems, colorimetry aids for accurate color notation and communication is an established field in the textile industry [37]. For color specification of a defined palette, the color of swatches, known as standard or pattern colors, are measured in a spectrophotometer, and their colorimetric properties are applied for the development of color reproduction based on a colorant database previously created [38,39]. The evaluation between the defined pattern color and the color samples dyed or printed with the formulated recipes involves comparative visual analysis and quantification of color differences, which can be subjective to acceptable tolerance limits [40].

Color biomimetics is a relevant topic in textile design, and the mimicking of natural pigmentary colors is commonly conducted with the aid of nature images or a selection of visual swatches that resemble a particular color. Instead of relying on visual references, this research explores the intersection of biomimetics and colorimetry by applying instrumental and systematic procedures directly with four selected natural plants to capture and reproduce their pigmentary colors.

The experimental work involved dyeing two polyester substrates with disperse dyes of different colors and concentrations using the exhaustion process. The dyed textiles were measured in a spectrophotometer, and a database was created in Datacolor Match Textile software with the samples’ colorimetric properties. The color of four natural plants was measured and set as a pattern color for the formulation of colorant recipes. After textile dyeing with recipes yielding the lowest expected color difference (ΔE*), a comparative analysis was conducted by spectral reflectance and visual assessment. Scanning electron microscopy and white light interferometry were also used to characterize the surface of the natural elements.

Therefore, this research successfully proposes a method for biomimicry pigmentary colors from natural plants in fibrous substrates and highlights the importance of colorimetry in textile design, allowing the reproduction of nature’s color palette through the effective use of colorants.

## 2. Materials and Methods

Two plain weave polyester (PES) substrates were used in the experimental work. Substrate 1 (S1) is a PES fabric with an optical brightener, has 101 g/m^2^, 18.0 Tex warp and weft yarns, and a density of 25 ends/cm and 26 picks/cm. Substrate 2 (S2) is conventionally manufactured in the textile industry, has 80 g/m^2^, 5.1 and 7.6 Tex warp and weft, respectively, and a density of 49 ends/cm and 36 picks/cm.

Samples were dyed with the following disperse dyes from the GOLDEN TECHNOLOGY brand (São Bernardo do Campos, Brazil): yellow SE-G; disperse blue SE-3RT 110%; disperse blue E-R 150%, disperse yellowish brown S-2R 150%, disperse orange S-G, disperse marine SE-AR, disperse ruby S-G 175, disperse red SE-3B, and disperse violet S2R. The Goldlevel E-PES product (combination of detergent, dispersant, and equalizer), also from the GOLDEN TECHNOLOGY brand, was also used.

### 2.1. Textile Dyeing

Textile dyeing processes were carried out using the exhaustion method in the HT-IR DYER equipment from the company TEXCONTROL (São Paulo, Brazil), model TC 2200, with infrared (IR) heating.

Initially, a set of samples was dyed, comprising a defined concentration range for each dye color. The dyeing concentrations were 0.1%, 0.5%, 1.0%, 2.0%, 3.0%, and 4.0%, and the dyebaths were prepared with a bath ratio of 1:20. A concentration of 3% in relation to the weight of the material was used for the Goldlevel E-PES auxiliary. The pH of the solutions was adjusted when necessary, obtaining values between 4.5 and 5.

For sample dyeing, each solution was heated to 90 °C at a rate of 9 °C/min. Subsequently, it was heated to 130 °C at a rate of 2 °C/min, remaining at that temperature for 30 min. Next, the process was cooled to 80 °C with a gradient of 3 °C/min.

The dyed samples were washed in running water, dried at room temperature, and after, they went through a reduction clearing process, with the aim of removing the dye that did not react with the fiber [41]. The treatment encompassed an aqueous solution with 2 g/L of sodium hydroxide, 2 g/L of sodium hydrosulfite, and 2 g/L of Coloremulg EMG 441 detergent, and it was carried out in the same equipment used for dyeing, with a temperature of 80 °C for 10 min. After washing, the samples were rinsed in running water and dried at room temperature.

To facilitate color measurement in the spectrophotometer, all textile samples were ironed with a cloth over the surface to avoid direct contact of the iron with the fabrics, ensuring that the surface was completely smooth.

### 2.2. Spectral Reflectance

Sample colors were measured using DATACOLOR (Lawrenceville, GA, USA) model 500 spectrophotometer equipment, with the illuminant D65, specular reflectance included, and observer 10°. The measurement procedure with concentration samples was conducted with an aperture of 9.0 mm. Due to the small size of the mimicked natural elements, color measurement was conducted with an aperture of 6.6 mm, and the analysis of the textile colors with the formulated recipes followed the same measurement setup. All measurements took place at four different points on each sample. The analysis was conducted in the CIEL*a*b* system, where coordinate L* represents perceived lightness and correlates perfect white (100) and perfect black (0), coordinate a* represents red (+a*) and green (−a*) and b* represents yellow (+b*) and blue (−b*).

The determination of sample’s color difference was calculated according to Equation (1).

(1) $$∆E^{*}=\sqrt{{\left(∆L^{*}\right)}^{2}+{\left(∆a^{*}\right)}^{2}+{\left(∆b^{*}\right)}^{2}}$$

where ΔE* represents the total color difference; ΔL* the color difference of the L* coordinate, Δa* the color difference of the a* coordinate, and Δb* the color difference of the b* coordinate.

The K/S values represent color strength, meaning the higher the value, the more intense the color is. The results attained for the concentration samples of each dye were calculated according to the Kubelka–Munk Equation (2).

(2) $$K/S=\frac{{\left(1-R\right)}^{2}}{2R}$$

where K is the absorption coefficient, S is the scattering coefficient, and R is the % reflectance value. K/S was calculated with the sum of K/S values [42] of every 10 nm wavelength between 360 and 700 nm.

### 2.3. Instrumental Colorant Formulation

#### 2.3.1. Spectrophotometric Calibration Data

Initially, a colorant database was created in the spectrophotometer Datacolor Match Textile software. In order to formulate recipes for color reproduction, it is necessary to develop a colorant set containing color information about the dyes and textile substrate handled.

This process requires color measurement of the samples and filling in additional information in the software. Data introduced consisted of (i) textile substrate: 100% PES woven fabric and color measurement of each substrate (S1 and S2); (ii) dyeing process: temperature (130 °C), dye class (disperse), bath ratio (1:20); dyeing method (exhaustion); (iii) colorants: for each colorant type vs. color file, each group of concentration samples were measured and associated.

#### 2.3.2. Colorant Formulation Recipe

The dyeing recipes were formulated in the Datacolor Match Textile software, based on the previously created database. First, the color to be mimicked needs to be measured in the spectrophotometer and defined as pattern color. After, it is possible to select the dyes’ colors that the software will consider to formulate the recipe. Furthermore, it is possible to select the minimum and maximum number of dyes in the recipe, which, in this case, were from 1 to 3 dyes, and define the illuminant (D65). After this selection, the colorant recipe was calculated. The program offers different recipes with variable expected ΔE* values. In this work, the recipe with the lowest ΔE* value was always selected.

**Figure 1:**
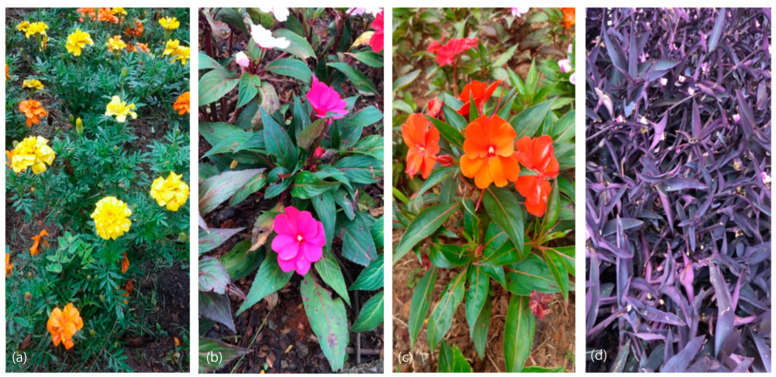
Natural elements defined for color biomimicry: (**a**) petal of yellow marigold flower, (**b**) petal of pink busy lizzie, (**c**) petal of orange busy lizzie, (**d**) leaf of purple trapoeraba.

Definition of the organic elements, which color be mimicked, comprised the four flowers/plants depicted in Figure 1: (a) petal of yellow marigold flower (*Tagetes*), (b) petal of pink busy lizzie (*Impatiens walleriana*), (c) petal of orange busy lizzie (*Impatiens walleriana*), (d) leaf of purple trapoeraba (*Tradescantia pallida purpurea*). The petals and leaves were subjected to measurements on the spectrophotometer to define their colorimetric properties.

### 2.4. Qualitative and Quantitative Analysis

A comparative analysis of the colors attained in the dyed samples with the formulated recipe and the natural plant colors was conducted by qualitative and quantitative methods. Visual assessment was carried out in an exterior environment under natural light. It is important to highlight that organic elements often exhibit non-uniform coloration across their petals and leaves. Therefore, the qualitative analysis focused on observing how textile colors blend with the dominant color tone of the plants. In addition, a photo record of the dyed textiles below each plant was recorded for research communication.

Spectral reflectance analysis included color measurement of the dyed textiles on the equipment detailed in Section 2.2. This analysis compared the colorimetric data of the dyed textiles with the corresponding plant pattern color they were developed to mimic.

To evaluate the surface morphology of the textile substrate samples, an analysis was conducted with the scanning electron microscopy JEOL brand (Musashino, Japan) model JSM-6390LV. Samples were initially deposited in a stub and covered with gold, using the “sputtering” technique in LEICA Camera AG brand (Wetzlar, Germany) EM SCD500 coating equipment.

To evaluate the surface of the flower’s petals and leaves selected, a professional Zoom 1000× MP digital microscope, NANYANG SRATE OPTICAL INSTRUMENT CO., LTD. brand (Nanyang, China), 8 LEDs with white light, was used.

White light optical interferometry is a technique that measures the phenomenon of interference of electromagnetic waves, generally in the wavelength of the visible spectrum, allowing to characterize the 3D shape and roughness of objects or surfaces [43]. This test was performed on the NewView 7300 interferometer, ZYGO brand (Middlefield, GA, USA), non-contact, three-dimensional, scanning white light interferometry, and MountainsMap software version 7.1 was used for data analysis.

## 3. Results and Discussion

### 3.1. Textile Dyed Concentration Samples

Textile samples dyed with the selected dye concentrations, ranging from 0.1 to 4% of each dye color, totalize 54 concentration samples for each textile substrate (S1 and S2). Color measurement was conducted in four different points of each sample, and the resulting mean value (considering ΔE* < 1) was applied in the development of the software colorants database.

#### 3.1.1. Colorimetric Properties of the Concentration Samples

Color coordinates L*, a*, and b* of the concentration samples dyed with the different dye colors and substrates are presented in Table 1.

For each color sample group, as the concentration of dyes increases, the L* value decreases. This is expected, precisely because the lower the L* value, the darker the sample. Furthermore, it is noted that for the yellow dye samples, there were high values of the b* coordinate. This is also justifiable since the increase in the positive value of this coordinate represents the yellow color. This also occurs for colors with yellowish brown and orange dye. The more negative the b* coordinate value, the greater the presence of the blue color. Dyes with the colors blue SE-3RT, blue E-R, marine, and violet showed high negative values. For the a* coordinate, the more positive the value, the greater the presence of the red color. This happened most clearly for the colors yellowish brown, orange, ruby, red, and violet. The more negative the values of the a* coordinates, the greater the presence of the green color.

Comparing the results attained with each dye color and concentration in the two different textile substrates, it was possible to analyze that S1, which is the substrate with an optical brightener, presents lower L* values in the majority of the samples, comparatively with S2, the substrate conventionally prepared, meaning that S1 samples are lighter than the corresponding S2 samples. This result was further analyzed with K/S values as following presented.

#### 3.1.2. Color Strength (K/S)

**Figure 2:**
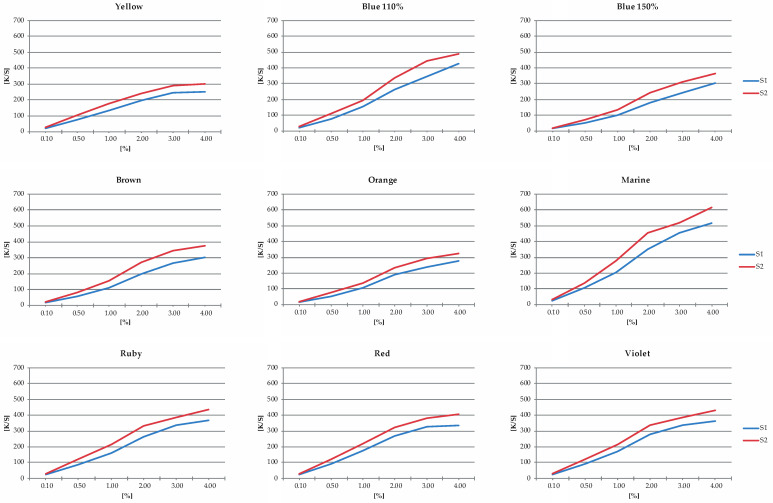
K/S of S1 and S2 substrates dyed with disperse dyes: Yellow, Blue 110%, Blue 150%, yellowish Brown, Orange, Marine, Ruby, Red, and Violet.

The K/S values were calculated with the aim of comparing the color strength of the samples and verifying the influence of the optical brightener on S1 in this property. Analyzing the images in Figure 2, a very similar behavior can be observed between all the dyes studied at different concentrations. S2 presented the highest color strength values in all samples’ colors and concentrations tested. This is justifiable due to the color of the textile substrates before the dyeing process, with S2 having the most yellowish color.

### 3.2. Biomimicry Color of Selected Flower Petals and Leaves

For color mimicking, the color of each flower petal and leaf selected for this study was measured in the spectrophotometer and set as pattern color in the instrumental recipe formulation process.

#### 3.2.1. Instrumental Color Recipe Formulation

Using the database developed in the Datacolor Match Textile software with the samples’ colorimetric properties of each dye color vs. substrate, instrumental dyeing recipes were formulated, setting the color of each flower petal and leaf to be reproduced as pattern color.

In the formulation process, the software presents several recipe options, considering different predicted ΔE* values. For each plant color vs. substrate, the recipe that presented the lowest ΔE* value was chosen (Table 2). The recipe for the yellow flower was only generated for S2 because, with S1, the recipe formulated with the lowest ΔE* presented 3.73 predicted value, which is expected to obtain a noticeable color difference. Recipes were only generated with ΔE* values equal to or less than 3. For this reason, to mimic the marigold flower yellow in S1, the recipe obtained for S2 was used. Furthermore, it is noted that the recipes for S2 presented a smaller amount of dye compared to S1 recipes. As previously discussed, this is due to the higher color strength results attained in S2, when using the same colorant concentration than in S1.

#### 3.2.2. Visual Analysis

By undertaking a comparative analysis with direct observation of the textile’s color and the natural plants, visual similarities can be assessed.

**Figure 3:**
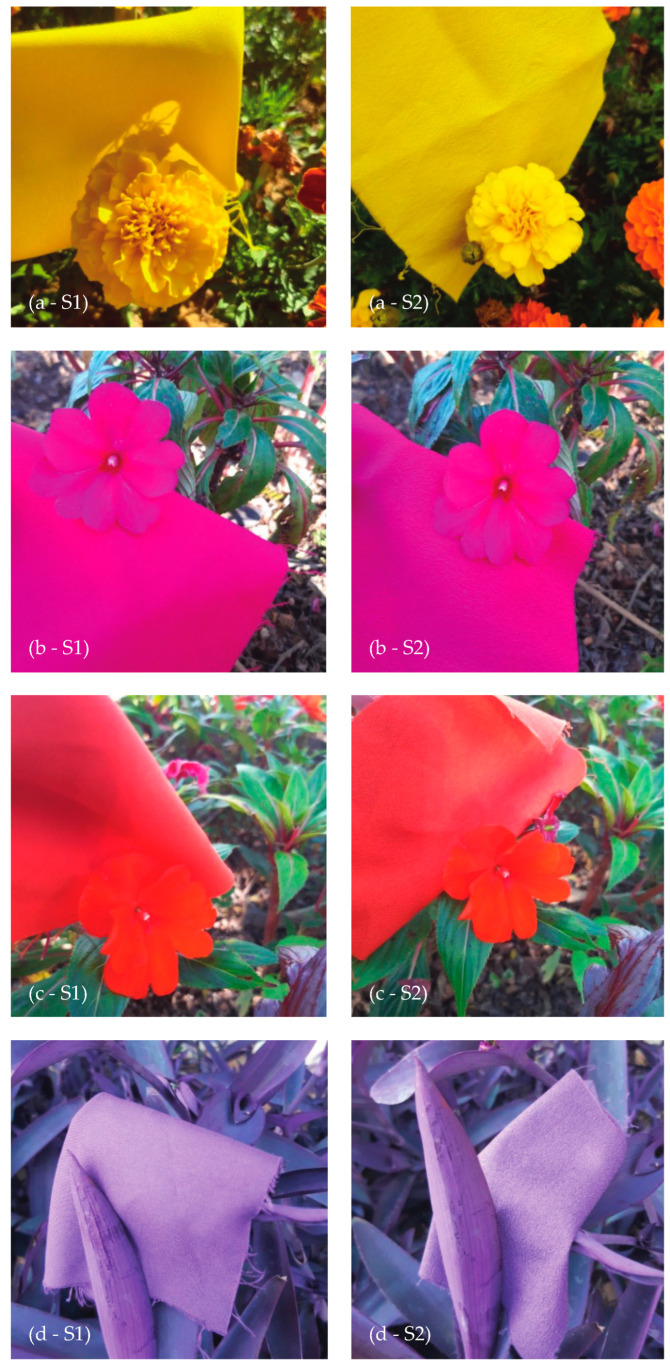
Photo of the dyed textile samples (S1 and S2) placed below the flowers’ petals and leaves aiming at color mimicry: (**a**) yellow marigold flower, (**b**) pink busy lizzie, (**c**) orange busy lizzie, (**d**) purple trapoeraba.

The dyeing of the samples carried out based on the colorimetric properties of the flowers’ petals and leaves, obtained good results visually, as observed in the photos presented in Figure 3. The yellow, pink, and purple colors of the natural plants were perceived with great similarity with the textile colors of both substrates. The most noticeable difference is for the dyeing of the orange color, mainly with S2, which presents a lower saturation when compared to the color of the biomimetic flower.

#### 3.2.3. Spectral Reflectance Analysis

For a quantitative analysis of the results, CIELAB color coordinates L*a*b* of each textile sample and natural element were compared, and the total color difference of each textile sample in relation to the corresponding pattern sample was analyzed (Table 3). As the measurement of the pattern colors was conducted with the aperture of 6.6 mm, due to the small size of the mimicked natural elements, the analysis of the dyed textile colors followed the same measurement setup.

Although great similarity was visually perceived between textile samples and the mimicked plants, the quantitative analysis highlighted differences, particularly in the yellow reproducing the color of the marigold flower with ΔE* = 9.8 (Sample A–S1) and 4.9 (Sample A–S2). The color of these samples presents high L* values, thus reporting light colors. Perception of color difference depends on several factors, including hue, saturation, and lightness. When analyzing light colors, differences in hue and saturation are less pronounced compared to more saturated or darker colors, with yellow hues being particularly challenging [44]. This can explain the variation between the quantitative and visual assessments.

Samples B, C, and D present ΔE* between 1.5 and 4.0 in comparison to the respective pattern colors. These results highlight the relevance of colorimetry in biomimetics of pigmentary colors, showing the ability to obtain good similarity with the colors of selected petals and leaves in the first formulated recipe, results that would be challenging to achieve by experimental mixing methods. Additionally, recipe optimization can also be conducted based on the colorimetric evaluation.

### 3.3. Scanning Electron Microscope and Optical Microscopy Analysis

**Figure 4:**
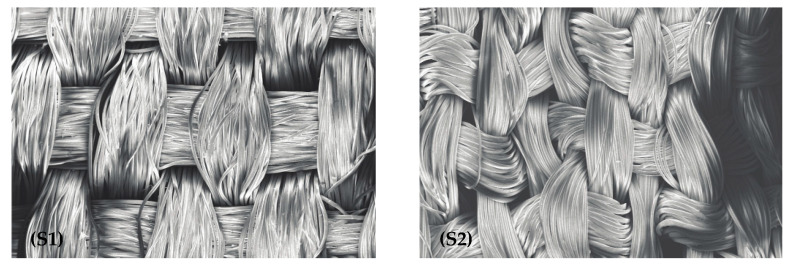
SEM images: magnified (100×) of (**S1**) and (**S2**), left and right, respectively.

**Figure 5:**
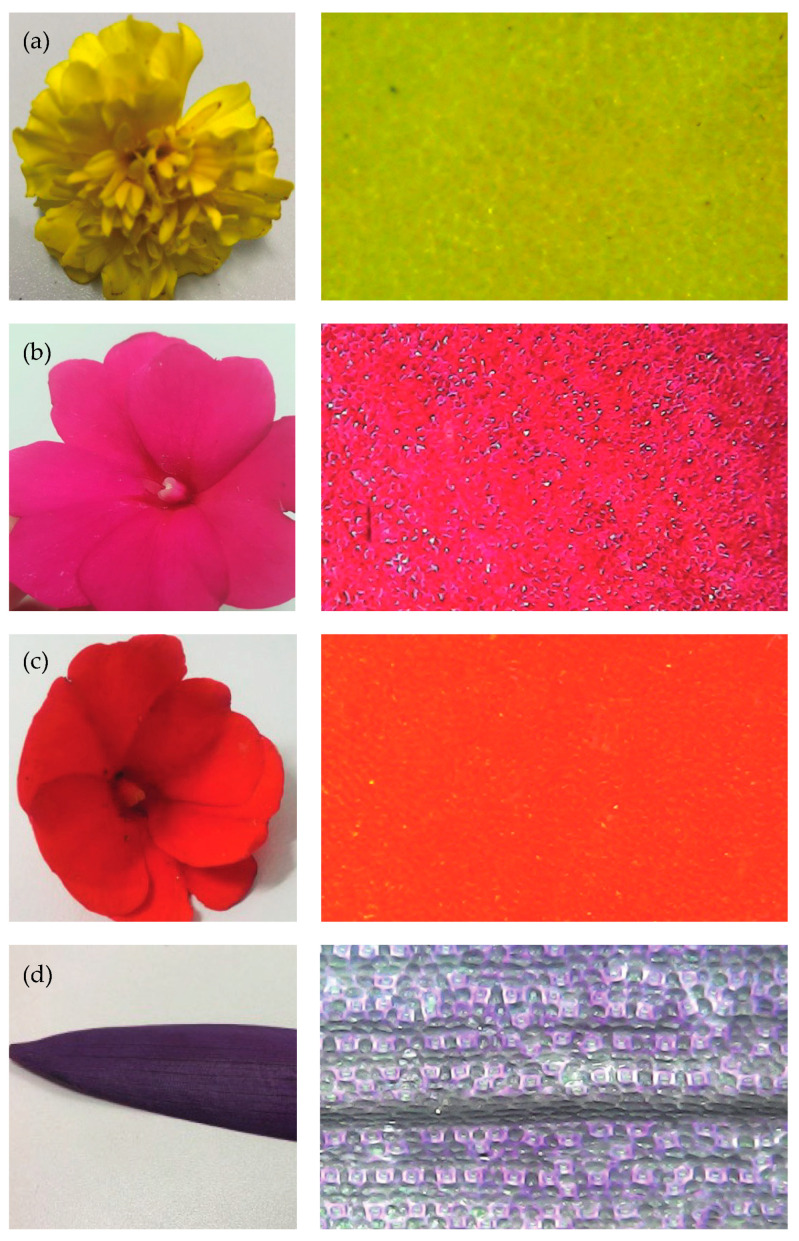
Image of the natural elements (left) and respective magnified surface 1000× (right): (**a**) yellow marigold flower, (**b**) pink busy lizzie, (**c**) orange busy lizzie, (**d**) purple trapoeraba.

Figure 4 presents the images of the textile substrates S1 and S2 with a magnification of 100×. Figure 5 shows the image of each natural element on the left and the magnified image (1000×) on the right.

Color is observed due to the reflection of the incident light that falls on a given object. Therefore, the structure of the reflective surface interferes considerably with the perceived color [33]. The textile substrates S1 and S2 have distinct surfaces, and the ΔE* variation observed for each color group may be related to the difference in surface roughness and the structure between the fabric and natural materials. If the surface is smooth, the specular reflectance component stands out; if it is rougher, the diffuse reflection will be more prominent [36]. Therefore, this factor can also influence the colorimetric values provided by the spectrophotometer.

Other factors that may also influence the color difference between the natural elements and the dyed textile can be related to morphology, structural uniformity, chemical components, humidity, and thickness, among others.

Given the non-uniform chromatics and complex surface morphologic properties that natural plant petals and leaves often exhibit, future work should encompass the measurement of multiple specimens of each plant to define pattern colors, as well as the development of a set of dyed samples for each pattern color.

### 3.4. White Light Interferometry

White light interferometry analysis was performed on all natural samples. However, a positive result was obtained only on the purple leaf, as the flower petals were quite thin and fragile, allowing light to pass easily through the samples.

**Figure 6:**
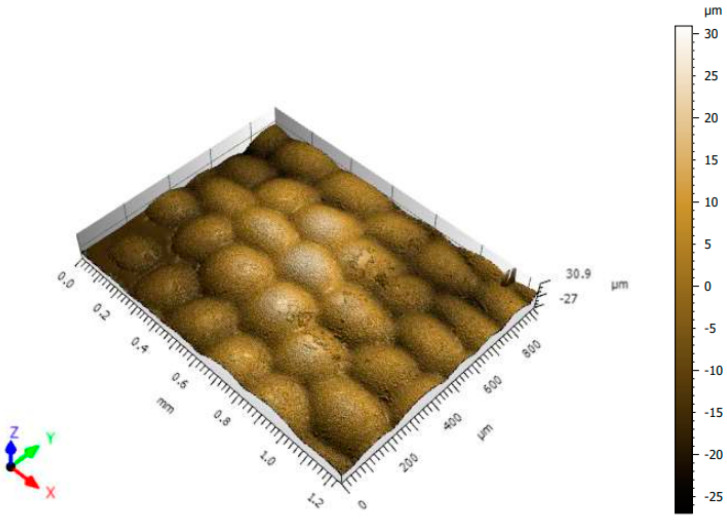
Topographic profile of the purple trapoeraba leaf.

In Figure 6, it is possible to observe that the purple leaf has a characteristic roughness on its surface. It should also be noted that the purple leaf has a structure that closely resembles taffeta, which is the textile woven pattern of the fabrics studied. However, the leaf does not have a superficial color homogeneity. The total color difference obtained in the previous results can also be influenced by the topographic difference between the textile substrates and the natural elements.

Based on colors from nature, designers can define a color palette through a creative exploration of colorimetry and biomimetics in textile design. The research conducted demonstrated how organic elements can be used throughout the complete design process, contributing to effective color reproduction and use of colorants.

**Figure 7:**
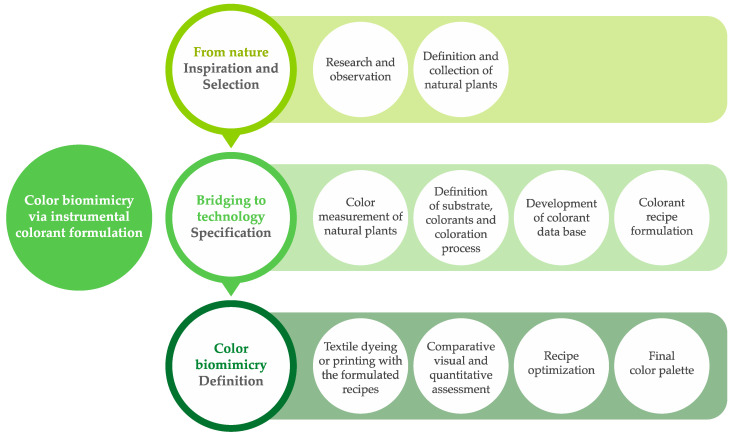
Design framework for biomimicry colors in textiles via instrumental colorant formulation.

A design framework was created, which sets a structured guide to the main phases and steps toward biomimicry colors in textile design via instrumental colorant formulation (Figure 7).

The framework depicts three main phases of the color palette definition through the biomimicry process. Initially, under the title ‘from nature’, the focus relies on color inspiration and color selection, involving research and observation in the first step, following the definition and collection of organic elements examples. This process requires designers to establish a relationship between the design concept, color theory, and natural colors selected. The second phase focuses on bridging nature and technology in color specification. The color of the selected elements has to be measured in a spectrophotometer, and the designer has to choose the parameters desired for textile coloration, namely textile substrate, colorant type, and coloration process. For instrumental colorant formulation, it is necessary to have a database with the colorimetric properties of the colorants selected and set each natural color as a pattern color to calculate and select the recipe with the lowest predicted ΔE*. The last phase finalizes the color biomimicry process and the final definition of the chromatic palette. Samples are dyed or printed with the formulated colorant recipes, and they are assessed through quantitative and qualitative methods using the natural elements selected. If necessary, to improve color similarity, fine-tuning of the recipes can be conducted based on analysis of samples’ colorimetric properties and the software correction function. Finally, the color palette is defined.

The intersection of colorimetry and biomimetics in textile design involves combining principles and methodologies from each area to accurately mimic pigmentary colors from nature, and the framework aims to contribute to the design process with a structured guide. Nevertheless, the framework is an initial attempt to integrate nature pigmentary colors into textile design through a systematic approach, presenting the possibility to evolve with more research development.

## 4. Conclusions

This research presents a method for biomimicry pigmentary colors in dyed textiles based on the spectral reflectance of natural elements and instrumental formulation of colorant recipes.

Textile samples dyed with the formulated recipes attained good chromatic similarity with the respective natural plants’ colors, and the majority of the samples presented ΔE* between 1.5 and 4.0. Additionally, recipe optimization can also be conducted based on the colorimetric evaluation.

The results attained demonstrate that by using a scientific and data-driven approach, designers can emulate with great accuracy nature’s color palette, being also a process aligned with sustainable practices, encompassing an efficient use of the colorants.

This research contributes a design framework proposed to guide the color biomimicry design process based on colorimetry and color theory, which encompasses color inspiration, selection, specification, and final textile color palette definition. Throughout this process, designers can ally creativity and systematic methods to achieve desired chromatic qualities.

Looking ahead, the possibility of creating a database with colorimetric properties of natural dyes that enable textile coloration with primary colors and subtractive color synthesis opens up innovative future research to reproduce nature’s palette with natural colorants. Another important approach with great potential for surface design is to research the implementation of the proposed method for textile coloration through printing processes for the exploration of both color and pattern.

## Figures and Tables

**Table 1:** Color coordinates L*, a*, and b* of the concentration samples.

Color	Concentration (%)	Substrate 1 (S1)			Substrate 2 (S1)		
		L*	a*	b*	L*	a*	b*
Yellow	0.1	86.2	−6.6	55.8	86.7	−4.6	69.9
	0.5	83.3	−2.5	86.3	83.0	2.8	93.9
	1.0	81.4	0.8	95.0	80.8	6.4	100.1
	2.0	79.6	4.6	98.7	79.0	10.3	101.5
	3.0	75.4	9.3	95.3	74.6	17.4	96.9
	4.0	76.5	12.0	97.5	72.4	19.5	93.5
Blue 110%	0.1	65.2	3.8	−32.3	57.5	4.8	−28.7
	0.5	45.1	6.8	−40.0	38.8	6.0	−35.3
	1.0	35.2	9.7	−41.1	30.9	8.8	−36.1
	2.0	27.5	12.4	−38.9	23.8	10.7	−33.2
	3.0	23.6	13.4	−36.1	20.2	11.1	−29.6
	4.0	20.9	13.3	−32.6	18.9	10.4	−26.1
Blue 150%	0.1	70.9	−3.2	−30.8	66.5	−6.3	−28.1
	0.5	53.7	−3.1	−38.8	47.2	−1.8	−36.9
	1.0	44.6	−1.0	−40.9	39.0	0.7	−38.5
	2.0	36.3	2.8	−41.3	31.0	4.6	−38.2
	3.0	31.5	5.9	−40.8	26.9	7.0	−36.8
	4.0	27.9	8.0	−39.2	24.2	8.5	−34.8
Yellowish brown	0.1	77.5	12.7	25.1	75.4	18.9	36.2
	0.5	65.0	24.6	43.5	61.2	29.7	46.9
	1.0	57.7	29.4	48.0	53.3	33.7	48.7
	2.0	50.3	33.4	48.1	45.2	35.5	45.3
	3.0	45.6	35.9	45.2	41.1	36.6	41.7
	4.0	42.5	35.8	41.5	38.5	36.7	37.8
Orange	0.1	78.1	15.6	26.0	77.7	22.2	39.3
	0.5	68.5	30.8	50.7	65.2	37.0	55.2
	1.0	62.2	38.5	58.3	61.2	44.2	62.6
	2.0	57.0	44.8	61.0	55.9	49.6	63.3
	3.0	55.1	48.8	61.8	52.4	51.7	60.6
	4.0	52.2	49.9	58.9	50.8	53.7	58.8
Marine	0.1	62.6	−0.9	−23.6	56.1	−2.2	−19.7
	0.5	39.4	−1.0	−25.7	34.9	−0.8	−22.0
	1.0	30.0	0.3	−24.3	25.3	0.8	−20.4
	2.0	22.4	2.3	−19.8	19.2	2.1	−15.8
	3.0	19.2	2.8	−15.8	17.7	2.4	−12.0
	4.0	17.8	2.8	−12.7	16.1	2.3	−8.6
Ruby	0.1	62.8	38.6	−8.5	59.3	43.8	−1.7
	0.5	46.2	50.9	−0.2	42.3	52.0	4.3
	1.0	40.0	53.0	5.5	36.5	52.1	8.2
	2.0	34.1	50.9	10.2	31.1	48.8	12.2
	3.0	30.9	48.0	12.6	28.8	45.3	13.7
	4.0	28.9	44.4	12.6	26.9	42.4	14.1
Red	0.1	64.0	44.8	−11.1	60.7	49.5	−4.3
	0.5	48.6	59.2	−1.4	45.7	60.3	3.7
	1.0	42.3	60.9	5.4	39.5	60.1	9.3
	2.0	37.3	58.7	11.8	34.7	56.5	14.7
	3.0	34.4	55.7	15.2	32.0	52.9	16.9
	4.0	33.2	52.7	16.2	30.7	50.3	17.7
Violet	0.1	59.9	21.9	−34.2	53.8	22.7	−31.3
	0.5	39.2	30.4	−38.5	34.3	29.4	−34.9
	1.0	30.3	32.1	−37.2	26.8	29.4	−32.6
	2.0	23.8	29.4	−31.9	21.2	25.8	−27.0
	3.0	21.2	26.2	−27.5	19.5	21.7	−22.1
	4.0	20.4	24.2	−25.0	18.3	19.8	−19.8

**Table 2:** Colorant recipes.

Dye Color	Pattern Color A: Yellow Marigold		Pattern Color B: Pink Impatiens		Pattern Color C: Orange Impatiens		Pattern Color D: Purple Trapoeraba	
	Sample A		Sample B		Sample C		Sample D	
	S1	S2	S1	S2	S1	S2	S1	S2
Yellow	1.524	1.524			0.457	0.259	0.201	0.092
Blue 110%							0.591	0.424
Blue 150%								
Yellowish brown								
Orange								
Marine								
Ruby								
Red			1.463	0.984	0.832	0.610	0.355	0.238
Violet					0.013	0.008		
Predicted ΔE*	2.4	X	0.6	0.9	0.1	0.0	0.0	0.0

**Table 3:** L*a*b* values of pattern color and dyed samples in S1 and S2, and ΔE*of each textile sample in relation to the correspondent pattern sample.

Natural Element	Sample	L*	a*	b*	ΔE*
Yellow marigold	Pattern color A	82.5	8.2	109.4	_
	Sample A–S1	81.7	3.6	100.8	9.8
	Sample A–S2	81.5	9.5	104.8	4.9
Pink impatiens	Pattern color B	38.6	61.4	8.9	_
	Sample B–S1	39.1	60.0	8.6	1.5
	Sample B–S2	39.3	59.1	8.7	2.4
Orange impatiens	Pattern color C	45.9	59.2	43.2	_
	Sample C–S1	45.0	56.0	41.0	4.0
	Sample C–S2	44.6	56.3	42.0	3.4
Purple trapoeraba	Pattern color D	29.5	11.2	−9.0	_
	Sample D–S1	30.0	13.1	−9.9	2.2
	Sample D–S2	31.0	12.2	−12.0	3.5

## Data Availability

The data presented in this study are openly available in Repositório Institucional da UFSC, https://repositorio.ufsc.br/handle/123456789/203245, accessed on 4 May 2024.
